# The effect of acute, moderate intensity indoor cycling on the temporal resolution of human vision system, measured by critical fusion frequency

**DOI:** 10.14814/phy2.14618

**Published:** 2020-11-12

**Authors:** Karina Maciejewska, Aleksandra Greń, Aleksandra Wieczorek

**Affiliations:** ^1^ Faculty of Science and Technology Institute of Biomedical Engineering University of Silesia in Katowice Chorzow Poland; ^2^ Faculty of Science and Technology University of Silesia in Katowice Chorzow Poland

**Keywords:** critical fusion frequency, flicker, physical effort, visual field, visual temporal resolution

## Abstract

Critical fusion frequency (CFF) reflects the basic temporal function of the visual system and therefore is a good measure of its performance. CFF has been implemented in psychological and pharmacological studies to evaluate cognitive functions. The influence of abnormal environmental conditions, such as physical exercise, has been recently explored. Previous studies have presented alterations of cognitive processes due to acute exercise. However, the duration of the effect after the end of exercise has not been investigated. This evaluation is important especially in reference to long‐term conclusions on the effect of training on CFF as an improvement of cognition. The main goal of this study was to check whether a stimulatory effect of acute submaximal physical exercise on CFF among non‐experienced cyclists persists over time. Moreover, we asked whether this effect differs between areas of visual field. CFF thresholds from 15 volunteers were measured by means of an automated medical perimeter PTS 910 (Bogdani) before, immediately after the end, and 30 min after the end of two sessions (training and rest). During rest, CFF did not change significantly, but we observed an increased CFF immediately after training. Interestingly, this increase was maintained 30 min after the end of exercise in fovea. A greater decrease of CFF during rest was observed for lower than for upper hemifield. Our results suggest that an acute, moderate‐intensity cycling improved CFF in non‐experienced cyclists, with the duration of the effect depending on eccentricity. The possible visual hemifield asymmetries of CFF changes over time will be further investigated.

## Graphical abstract

CFF thresholds from 15 volunteers were measured before, immediately after the end, and 30 min after the end of two sessions (training and rest). We observed an increased CFF immediately after training. Interestingly, this increase was maintained 30 min after the end of exercise in fovea.

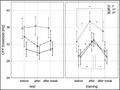

## 1 INTRODUCTION

Critical fusion frequency (CFF), also known as critical flicker fusion frequency (CFFF) or flicker fusion frequency (FFF) reflects the basic temporal function of the visual system and therefore is a good measure of its performance (Eisen‐Enosh et al., 2017; Schrupp et al., 2009). It can be determined in two ways. In the first one, the measurement starts with a light flickering at such a high rate that it is perceived as a steady light and then the frequency is decreased (flicker mode). In the second mode, the measurement starts with a light flickering at such a low rate that it is perceived as a flickering light and then the frequency is increased (fusion mode). The participant is asked to point (e.g., by pressing a button) when the light starts to flicker in the flicker mode or when the light stops to flicker in the fusion mode. In both cases, CFF threshold is regarded as the threshold frequency (measured in Hz) at which the perception of the flickering light is changed. The higher CFF value means a better temporal resolution.

Critical fusion frequency has not only been known as an objective, quantitative, and important measure of retinal function. CFF has also been implemented in psychological and pharmacological studies to evaluate cognitive functions, such as sensory sensitivity, information processing, perceptual load, anxiety level, alertness and cortical arousal, CNS fatigue, response to emotional stimulation, the effect of age, or physical exhaustion and fatigue (Clemente‐Suárez & Diaz‐Manzano, 2018; Dustman et al., 1984; Godefroy et al., 2002; Lambourne & Tomporowski, 2010; Lawson et al., 2014; O'Brien et al., 2017; Saint et al., 2017; Simonson & Brozek, 1952; Truszczyński et al., 2009). CFF has recently become an interesting tool used in exercise science research to explain physiological and pathological stress situations, such as physical exercise or environmental changes. However, due to differences in investigated protocols, types of activity, and its duration, it is often very difficult to compare the results and draw long‐term conclusions based on the previous findings.

Recently Hanson et al. (2018) determined the effect of exercise type and intensity on neural arousal by means of CFF among recreational runners. The results suggested that short, fatiguing exercise affects cortical neural arousal differently than longer, steady‐state training. Increases in arousal, and perhaps the related domain of information processing, are more likely to come from steady‐state exercise at a vigorous intensity.

Lambourne and Tomporowski (2010) in their meta‐analysis based on 29 studies that provided pre‐ and post‐exercise cognitive measures showed that participants' cognitive performance improved when tested after exercise. The results confirmed the earlier predictions that metabolic recovery occurs gradually and that the heightened level of arousal during this period facilitates cognitive function. At the same time, the authors emphasized the lack of studies presenting changes following the termination of exercise and the need for investigating short‐term after‐effects.

The study by Hanson et al. (2018) is important because it emphasizes that cortical neural arousal is differently affected by the type, intensity, and duration of physical exercise. However, it does not explore the duration of the effects caused by physical exercise. This knowledge is extremely important especially in the light of the possible beneficiary effect of training on the processing of visual stimuli as an improvement of cognition, training procedures or sport strategies. The meta‐analysis performed by Lambourne and Tomporowski (2010), in turn, underlines the need for investigating the duration of the observed effects. Our study fills the gap in this area.

Moreover, further investigation of the possible differences in the impact of physical exercise on CFF across the VF (visual field) is needed. The asymmetries related to eccentricity, as well as vertical and horizontal VHs (visual hemifields) in many aspects of the processing of visual stimuli, have been widely discussed (Al‐Nimer & Al‐Kurashy, 2007; Anderson & Vingrys, 2002; Carlei & Kerzel, 2017; Cheng et al., 2019; Corbetta & Shulman, 2011; Curcio & Allen, 1990; Levine & McAnany, 2005; Powell, 1982; Rezec & Dobkins, 2004; Schrupp et al., 2009; Silva et al., 2014; Simonson & Brozek, 1952; Walter et al., 2015; Woods & Thomson, 1995; Wright et al., 2016; Zito et al., 2016). However, to our best knowledge, the literature lacks studies investigating the duration of the effect of acute exercise on the temporal resolution of human visual system, taking into account differences between eccentricities, horizontal VHs and vertical VHs.

Therefore, our study aimed at answering three scientific questions. The first goal was to investigate the duration of a stimulatory effect of acute submaximal physical exercise among non‐experienced cyclists. If the stimulatory exercise‐induced effect persists over time, it will have a great value in terms of human visual processing.

The second goal was to answer a question of whether this effect differs between central and peripheral VFs. Since VF is not uniform and the dynamics of different visual pathways differ, we should observe differences in the changes in CFF between areas of VF.

Third, possible differences between VHs in visual processing have been previously discussed in terms of differences in spatial attention, structural organization, or cortex asymmetries. However, there is a high inconsistency in these findings. Therefore, we compared the results between upper and lower, as well as left and right VHs.

## 2 METHODS

### 2.1 Ethical approval

Informed written consent was obtained from all participants included in the study. The procedures followed were in accordance with the ethical standards of the responsible committee on human experimentation (institutional and national) and with the Helsinki Declaration of 1975, as revised in 2013. The studies were approved by the Committee of Ethics of the University of Silesia in Katowice, Poland on scientific studies conducted on humans (number 3/2018). All mandatory laboratory health and safety procedures have been complied within the course of conducting any experimental work reported in this paper.

### 2.2 Participants

The experiment was conducted on the right eyes from 15 volunteers at the age 23.9 (1.0) years (8 females). Information about health condition and lifestyle of the participants was gathered in a questionnaire. All the participants fulfilled the inclusion criteria: had normal color perception and normal acuity (the sight defects were all below ± 0.5 D), were healthy, non‐smokers, with no ophthalmological, neurological or cardiological medical history, and did not exercise regularly. None of the participants had consumed alcohol, medicaments, drugs, intoxicants or other substances that may affect cognition within 24 hr prior to the study.

### 2.3 Apparatus

Critical fusion frequency examinations were conducted by means of an automated medical perimeter PTS 910 (Bogdani, Poland). Green LED light stimuli (565 nm) of Goldmann III size and maximal intensity (1,000 asb) were presented on a concave dome with the white background luminance of 10 Asb (±20). The HR was recorded with the use of Onrhythm 500 heart rate monitor (Geonaute) and the training was performed on New‐Wave Bike I cycling bike (Besmarex).

### 2.4 Procedure

**FIGURE 1:**
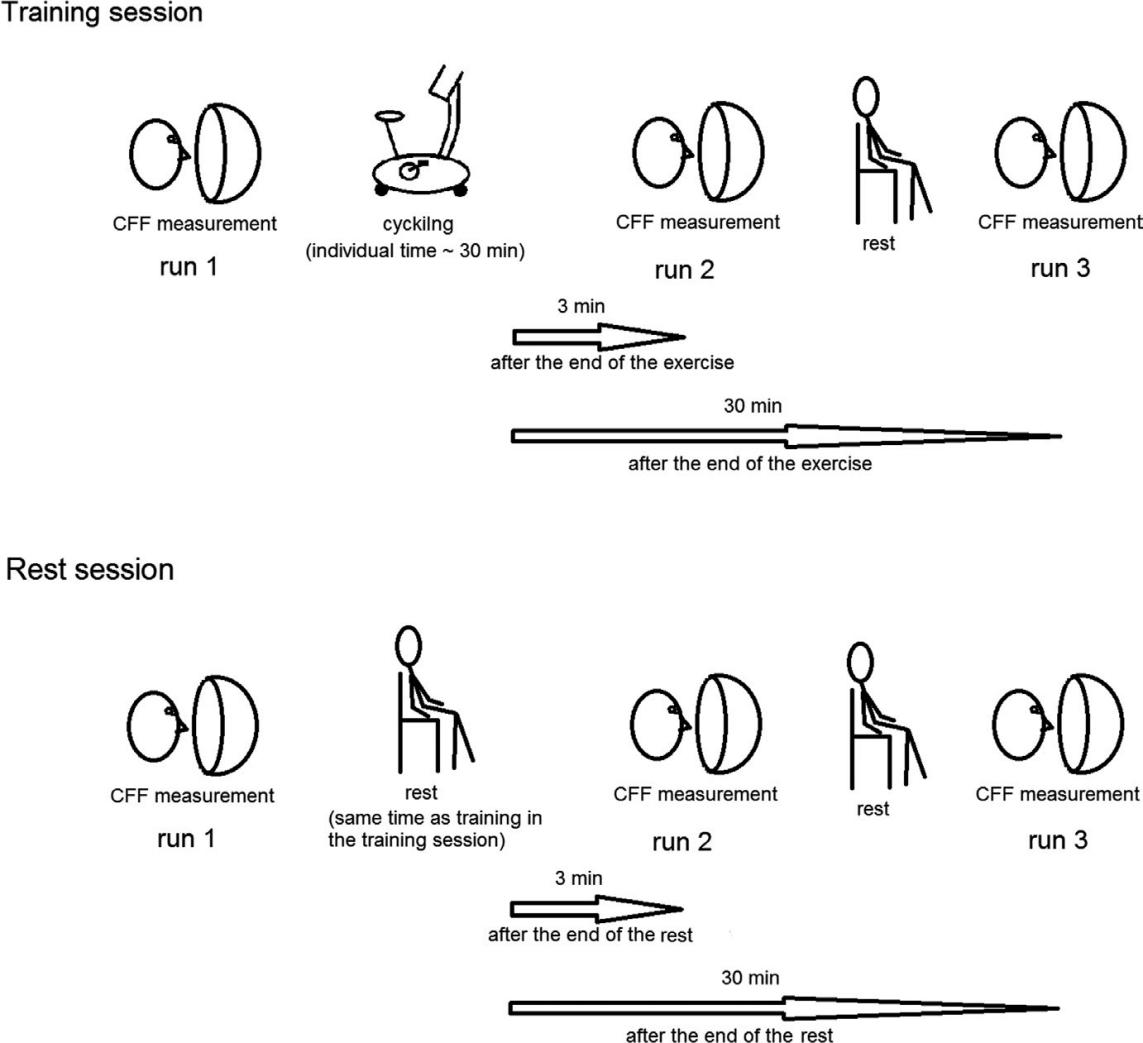
The experimental design. The experiment was conducted in 2 days and consisted of two sessions: training (indoor cycling) and rest session. The training session was conducted on the first day, followed by the rest session on the other day. The participants conducted critical fusion frequency (CFF) measurement at three phases defined in relation to the exercise: before the start of the cycling exercise (run 1), immediately (i.e. including 3 min of dark adaptation) after the end of the cycling exercise (run 2), and 30 min after the end of the cycling exercise. In the rest session, the participants were seated in the same environmental conditions as during cycling and did not perform any physical activity. They conducted CFF measurement in the same way as in the training session at three phases defined in relation to the duration of the cycling exercise in the training session

The experiment was conducted in 2 days and consisted of two sessions: training and rest sessions. The training session was conducted on the first day, followed by the rest session on the other day. Before the training session, participants were given instructions about tasks and a practice session. The aim of the practice session was to introduce the participants with the measurement and to minimize the number of errors during the following sessions. After a predefined error criterion was reached, the training session was conducted. The experimental design is presented in Figure 1.

In the training session, the participants performed indoor cycling. We chose this type of training, because: (a) it is an aerobic exercise aiming at developing both cardiorespiratory endurance and body composition, (b) is commonly used for submaximal exercise testing, and (c) requires minimal skill or physical fitness to perform (Heyward & Gibson, 2014). Cardiorespiratory endurance is the ability to perform dynamic exercise involving large muscle groups at moderate‐to‐high intensity for prolonged periods. Body composition is a key component of an individual's health and physical fitness profile. The cycle ergometer is easy to instrument and is the preferred modality for exercise tests conducted on individuals with conditions affecting their ability to safely walk or jog on a treadmill (Balady et al., 2010). Moreover, a recent meta‐analysis showed larger effect sizes associated with cycle ergometry when compared to the treadmill (Lambourne & Tomporowski, 2010). These differences were speculated by the authors to arise from differences in cortical activation specific to the musculature involved in each task. Therefore, due to the fact that the previous literature on the impact of physical effort on human vision is not consistent in terms of the type of exercise, workload, and the level of participants' experience, we chose cycling as the most commonly used protocol giving the largest effect sizes.

The participants conducted CFF measurement (see below) at three phases defined in relation to the exercise: before the start of the cycling exercise (run 1), immediately after the end of the cycling exercise (run 2), and 30 min after the end of the cycling exercise. During the whole experiment, the participants had the heart rate monitor on. Initial HR value (before the start of the cycling exercise) was recorded until it stabilized and when a stable hemodynamic condition was achieved, HR was noted (approximately 30 s, Palatini et al., 2006; Palatini & Julius, 1997; Vogel et al., 2004). The duration of cycling was defined for each participant individually as that during which the predefined relative workload was reached and maintained for 20 min. The use of individual relative workload is recommended in exercise psychology research (Davranche & Audiffren, 2004).

Regarding the workload, we employed 75% of maximal aerobic power (75% of HR_max_ calculated as HR_max_ = 220 [bpm] – a, where a is the age of the person). Submaximal tests assume that the HR_max_ for people of a given age is similar, so this value is estimated from age. The aforementioned equation is widely used (Heyward & Gibson, 2014). HR during exercise has been studied widely in humans and is one of the methods used in training programs to estimate the intensity of the exercise performed (McGowan & Hampson, 2016). HR_max_ adjusted for age is required to elicit desired aerobic exercise intensity (Brown et al., 2006). An exercise intensity of 50%–85% of the maximal oxygen uptake (VO_2_max) is recommended for exercise programs designed to improve cardiorespiratory endurance. Heart rate is linearly correlated with oxygen uptake, so the estimation of exercise heart rates equivalent to 50%–85% of VO_2_max can be calculated to estimate an individual's exercise energy expenditure (Heyward & Gibson, 2014). A straight percentage of maximal HR (percent heart rate maximum, %HR_max_) to estimate exercise intensity and determine target exercise HR can be used because the %HR_max_ is related to %VO_2R_ (a difference between VO_2 max_ and VO_2 rest_) and %HRR (percent heart rate reserve used to determine target HRs for exercise training, where the heart rate reserve, HRR, is the difference between the maximal HR and resting HR). Exercise intensities of 74%–84%HR_max_ (55%–70%HRR or %VO_2_R) are equal to the average cardiorespiratory fitness classification and moderate‐hard workload in healthy adults (Heyward & Gibson, 2014). This training setup allowed us to obtain an acute, moderate intensity, effective training without physical fatigue caused by overtraining.

In the rest session, the participants were seated in the same environmental conditions as during cycling and did not perform any physical activity. They conducted CFF measurement in the same way as in the training session (see below) at three phases defined in relation to the duration of the cycling exercise in the training session (run 1, run 2, and run 3). The fact that the rest session was performed on a different day, which was later than the training day, allowed us to set the timing of CFF measurements the same as the timing of CFF measurements during the training session. The time interval between training and rest sessions was random (10 ± 9 days) to minimize any possible influence of the training session on the rest session. The tests were performed during the late morning or the early afternoon, for both rest and training sessions.

Before the first CFF measurement (run 1), stable HR was read from the heart rate monitor. After the first CFF measurements in both sessions, the participants were moved to a room next door, where the cycling exercise was performed. Both the cycling exercise in the training session and resting in the rest session were performed under normal illumination. After cycling exercise in the training session and the resting in the rest session, the participants were immediately moved back to the room, where the CFF measurements were taken. They were seated in front of the perimeter, the light was turned off for 3 min to allow for dark adaptation, during which stable HR was read from the heart rate monitor (in the same way as the initial HR). Due to the necessity of dark adaptation, when using the term “CFF measurement immediately after the training” in this work to describe the second CFF measurement (run 2), we have in mind this 3 min delay. After the second CFF measurement (run 2), the participants were seated under normal light condition again. Thirty minutes after the end of the training, participants were again seated in front of the perimeter. After 3 min of dark adaptation and HR measurement, third CFF measurement was performed (run 3).

### 2.5 CFF measurement

During CFF measurements, the volunteers were seated in a quiet room, in a comfortable position in front of the perimeter. The non‐examined eye was covered by a patch and the position of the examined eye was regulated toward the perimeter fixation camera. According to statistical guidelines for the analysis of data obtained from one or both eyes (Armstrong, 2013) and to avoid confounding the results by different timings of the CFF measurements, only one eye (right, selected a priori) from each participant was tested. The chin rested on the machine and a button was given to the volunteers to be used during the examination. Before each CFF measurement, a 3‐min long dark adaptation period was used in order to reach the CFF threshold plateau (Eisen‐Enosh et al., 2017).

The CFF examination begun with a calibration phase, where the frequency of the flashes was set to a beginning value (based on the mean CFF from 4 points at 10° eccentricity, and increased by 12 Hz). Then it decreased with a step of 4 Hz up to 3 Hz. Participants were instructed to click a button when they noticed a flickering. The lights were presented for 3 s or until the subject's reaction. Perimetry is a subjective method, so in order to ensure the reliability of the results, two types of errors were monitored: false positive and false negative errors. The first one occurred if the participants pushed a button when they should not have (the light did not flicker). The latter occurred if they did not push the button when the light flickered with a very small (5 Hz) frequency that should have been noticed by everyone. All the errors in the experiment were kept under 15% (warning level), according to the manufacturer's recommendations. A custom arrangement of 24 light stimuli was used, which shortened test duration to approximately 3–4 min. This is very important as previous reports show the long test duration introduces an effect of fatigue or loss of attention (Davranche & Audiffren, 2004; Eisen‐Enosh et al., 2017). In order to cover the whole VF evenly, four stimuli were presented at eccentricities: 3°, 10°, 15°, 22°, and 8 stimuli were presented at 30° eccentricity. Stimuli presented at 3°, 15°, and 30° eccentricity were included in the analysis, as a representation of: foveal (central), perifoveal, and midperipheral VFs, respectively. During each examination, pupil diameter was also measured.

### 2.6 Statistics

Statistical analysis was performed using Statistica 13.1 software (Dell). To verify the assumptions for the use of parametric tests, Shapiro–Wilk's test was used to check the normality of the data distributions in the analyzed groups and Levene's test to check variation homogeneity. The Greenhouse–Geisser correction for nonsphericity and post hoc comparisons with Bonferroni correction was used. For data which did not fulfill the normal distribution and variance homogeneity assumptions, non‐parametric tests were used. In all analyses, *p* < .05 was regarded as statistically significant.

Pearson's correlation was used to correlate CFF thresholds with biological factors. Repeated measures ANOVA was used for a comparison of participants' pupil diameters, as well as participants' HRs, between runs and sessions, with within‐subject factors: RUN (run 1, run 2, run 3), and SESSION (training, rest).

The comparisons of CFF between sessions, runs, eccentricities, and VHs were performed in three stages, in order to cover the three scientific questions we asked. To fulfill the first goal, CFF measured before, immediately after the end, and 30 min after the end of the training session were compared to results obtained during rest in time intervals matched to those in the training session. To answer the second question, we compared CFF changes between 3°, 15°, and 30° eccentricities, as a representation of foveal (central), perifoveal, and midperipheral VFs, respectively. To answer the third question, we compared CFF changes between upper and lower VHs, as well as between left and right VHs.

First, CFFs were compared between runs, sessions, and VHs for each eccentricity separately. Repeated measures ANOVA was used in this stage, with within‐subject factors: RUN (run 1, run 2, run 3), SESSION (training, rest), and VERTICAL VH (lower, upper) for vertical hemifields comparison and RUN (run 1, run 2, run 3), SESSION (training, rest), and HORIZONTAL VH (left, right) for horizontal hemifields comparison.

Second stage of the analysis was performed to compare the size of CFF changes in the training and rest sessions between analyzed eccentricities and VHs: horizontal and vertical. Differences in CFF thresholds between run 1 and run 2 (CFF_after‐before_) as well as between run 1 and run 3 (CFF_after break‐before_) were calculated. CFF_after‐before_ difference was calculated by subtracting the CFF values measured for run 2 from the CFF values measured for run 1. CFF_after break‐before_ difference was calculated by subtracting the CFF values measured for run 3 from the CFF values measured for run 1. Each difference was calculated for each stimulated point and each person separately.

CFF_after‐before_ and CFF_after break‐before_ differences were compared between sessions by means of Mann–Whitney *U* test with continuity correction with a factor SESSION (training, rest), ECCENTRICITY (3°, 15°, and 30°), VERTICAL VH (lower, upper), and HORIZONTAL VH (left, right).

For comparisons between eccentricities, Kruskal–Wallis test was used with a factor ECCENTRICITY (3°, 15°, and 30°) and SESSION (training, rest), for each CFF difference. For comparisons between vertical VHs, Mann–Whitney *U* test with continuity correction was used with a factor VERTICAL VH (lower, upper), SESSION (training, rest), and ECCENTRICITY (3°, 15°, and 30°), for each CFF difference. For comparisons between horizontal VHs, Mann–Whitney *U* test with continuity correction was used with a factor HORIZONTAL VH (left, right), SESSION (training, rest), and ECCENTRICITY (3°, 15°, and 30°), for each CFF difference.

In the third stage (exploratory analysis), two parameters were computed for each stimulated point and each person separately: CFF_(after‐before training)−(after‐before rest)_ and CFF_(after break‐before training)−(after break ‐before rest)_. Each of these two parameters was analyzed with a factor ECCENTRICITY (3°, 15°, and 30°), VERTICAL VH (lower, upper), and HORIZONTAL VH (left, right). Kruskal‐Wallis test was used for comparing CFF_(after‐before training)−(after‐before rest)_ and CFF_(after break‐before training)−(after break‐before rest)_ between eccentricities, and Mann–Whitney test with continuity correction was used for comparing them between hemifields.

## 3 RESULTS

**FIGURE 2:**
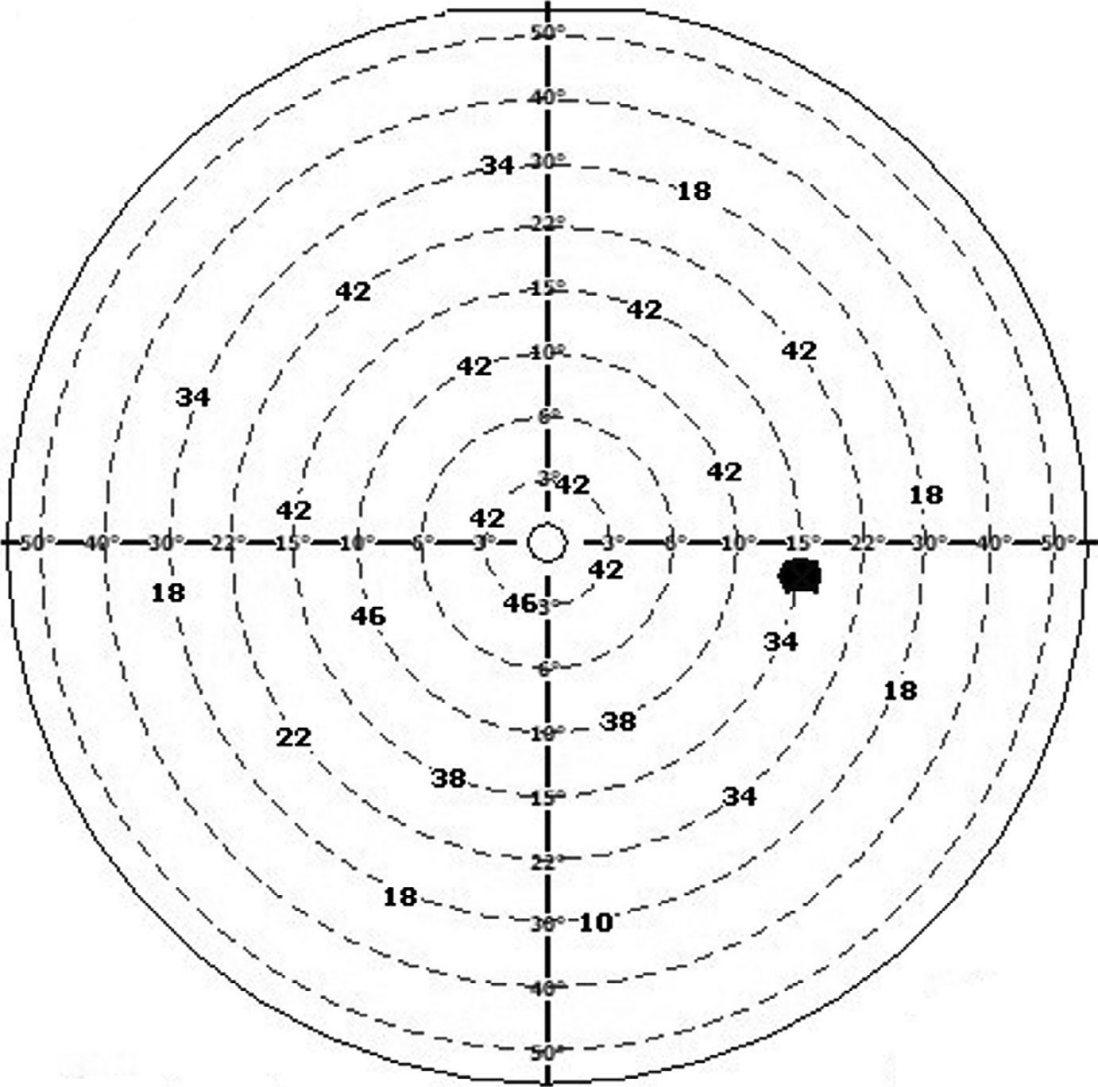
A representative map of absolute critical fusion frequencies [Hz] measured for one subject's eye, with the locations of 24 light stimuli presented over visual field

Figure 2 presents a representative map of the absolute critical fusion frequencies [Hz] of a representative subject's eye, with the locations of 24 light stimuli presented over VF: 4 points at eccentricities: 3°, 10°, 15°, 22°, and 8 points at eccentricity 30°. The CFF values obtained throughout three runs during training and rest sessions measured at eccentricities: 3°, 15°, and 30° were next included in the statistical analyses.

### 3.1 A control for biological factors influencing CFF

Critical fusion frequency depends on biological factors, such as age, body mass, pupil diameter, and psychophysiological condition. A negative correlation between the age of adults and the CFF has been reported in several independent studies and can be considered as a well‐established fact, interpreted as due to the narrowing down of the size of the pupil and the increased light absorption in the lens of older individuals (Simonson & Brozek, 1952). CFF has been also reported to differ between individuals of different body build, where the leptosomes had higher CFF values than the pycnics (Simonson & Brozek, 1952). Initial fatigue level differences must be ruled out as the possible psychophysiological confounding factor influencing the participants' ability to perform the task and distinguish the light stimuli from the background.

Therefore, we correlated CFF with age, weight, height, fatigue level, initial pupil diameter, and initial HR of the participants. None of the correlations of these factors with initial CFF thresholds was significant (Table 1).

**TABLE 1:** Correlations of initial critical fusion frequency (CFF) values with behavioral factors. None of the correlations was significant

Biological factor that is correlated with initial CFF	Mean value (*SD*)	Statistical results
Age (years)	23.9 (1.0)	*t*_15_ = 0.77, *p* = .45, *r* = .21
Weight (kg)	67 (15)	*t*_15_ = −0.86, *p* = .41, *r* = −.23
Height (cm)	173 (12)	*t*_15_ = −0.25, *p* = .81, *r* = −.07
Fatigue level	2.0 (0.9)	*t*_15_ = −0.07, *p* = .94, *r* = −.02
Initial pupil diameter (mm)	4.8 (1.4)	*t*_15_ = 0.6, *p* = .55, *r* = .17
Initial HR (bpm)	81 (12)	*t*_15_ = −0.13, *p* = .9, *r* = −.04

In order to assure the exercise in the training session induced the increase in participants' HRs and that this increase in HR was present when the second CFF measurement was taken (run2), we compared HRs between runs and sessions. Mean HR values for run 1, run 2, and run 3 were as follows: 86 (14) bpm, 81 (15) bpm, 82 (13) bpm in the rest session, and: 81 (12) bpm, 145 (8) bpm, 85 (11) bpm, in the training session. There was main effect RUN (*F*_2,56_ = 191, *p* < .0001), and an interaction effect RUN × SESSION (*F*_2,56_ = 235, *p* < .0001). Post hoc analysis revealed an increased HR in run 2 compared to run 1 only in the training session (*P*_run1‐run2_ < 0.0001, *P*_run2‐run3_ < 0.0001 and *P*_run1‐run2_ = 0.64, *P*_run2‐run3_ > 0.99 in the training and rest sessions, respectively). Moreover, at run 3, HR recovered to the values at run 1 in both rest and training sessions (*P*_run1‐run3_ > 0.99 and *P*_run1‐run3_ > 0.99 in training and rest sessions, respectively).

Critical fusion frequency increase after training may be influenced by exercise‐induced pupil dilatation, because wider pupil increases the illumination area of the retina (Gutherie & Hammond, 2004; Simonson & Brozek, 1952; Smith & Misiak, 1976). To account for that, we compared pupil diameter differences in training and rest sessions, which did not change significantly throughout runs either in rest: 4.44 (1.15) mm, 4.59 (1.17) mm, 4.72 (1.1) mm, or in the training session: 4.77 (1.40) mm, 4.92 (1.27) mm, 4.77 (1.34) mm. There was no main effect RUN (*F*_2,56_ = 0.8, *p* = .44), nor an interaction effect RUN × SESSION (*F*_2,56_ = 0.7, *p* = .48).

### 3.2 Comparison of CFF thresholds

Critical fusion frequency is related to the intensity of stimulus differently in central and periphery due to a decrease in ganglion cell density in receptive fields away from the fovea and the adaptation level of the ganglion cells with increasing eccentricity (Anderson & Vingrys, 2002; Schrupp et al., 2009; Simonson & Brozek, 1952). We did not use M‐scaling or F‐scaling to adjust for these effects, because our goal was to compare CFF thresholds between runs, which means all the spatial factors were kept the same in each run. We thus compared CFFs between runs (before training/rest, immediately after the end of the training/rest, and 30 min after the end of the training/rest) and sessions for each eccentricity separately. Mean CFF thresholds for each session, run, VH, and eccentricity are gathered in Table 2. Statistical results of this step of the analysis are in Table 3.

**TABLE 2:** Mean critical fusion frequency (CFF) thresholds for each session, run, visual hemifield (VH), and eccentricity with standard deviations (*SD*), confidence intervals at 95% (95% CI), and coefficients of variation (CV [%])

Eccentricity	VH	Mean CFF ± *SD* (95% CI)					
		Rest session			Training session		
		Run 1	Run 2	Run 3	Run 1	Run 2	Run 3
3°	Upper	35.7 ± 6.1 (33.5; 38.0), 17.0	36.7 ± 6.0 (34.4; 38.9), 16.4	36.1 ± 4.6 (34.4; 37.9), 12.7	32.8 ± 7.4 (30.0; 35.6), 22.5	37.5 ± 5.3 (35.5; 39.4), 14.1	35.2 ± 6.9 (32.6; 37.8), 19.7
	Lower	35.8 ± 5.7 (33.6; 37.9), 16.0	36.0 ± 4.9 (34.2; 37.8), 13.5	35.6 ± 5.2 (33.7; 37.5), 14.6	32.4 ± 7.4 (29.6; 35.2), 22.9	37.5 ± 5.9 (35.3; 39.7), 15.8	34.9 ± 6.0 (32.7; 37.2), 17.1
	Left	35.1 ± 5.6 (33.0; 37.2), 16.0	35.7 ± 5.0 (33.9; 37.6), 13.9	35.9 ± 5.1 (34.0; 37.8), 14.3	32.9 ± 6.8 (30.4; 35.5), 20.7	37.9 ± 5.8 (35.7; 40.0), 15.3	34.9 ± 5.9 (32.7; 37.1), 16.9
	Right	36.4 ± 6.1 (34.2; 38.7), 16.7	36.9 ± 5.9 (34.7; 39.1), 15.9	35.9 ± 4.7 (34.1; 37.6), 13.2	32.3 ± 7.9 (29.3; 35.2), 24.6	37.1 ± 5.4 (35.1; 39.1), 14.5	35.2 ± 7.0 (32.6; 37.8), 19.9
15°	Upper	33.1 ± 8.2 (30.0; 36.1), 24.8	32.8 ± 7.9 (29.8; 35.8), 24.2	33.7 ± 8.9 (30.4; 37.1), 26.5	29.2 ± 9.4 (25.7; 32.7), 32.1	33.2 ± 7.6 (30.4; 36.0), 22.8	30.9 ± 8.7 (27.7; 34.2), 28.1
	Lower	34.5 ± 7.7 (31.7; 37.4), 22.3	30.3 ± 7.9 (27.3; 33.2), 26.1	32.0 ± 6.7 (29.5; 35.5), 20.8	29.2 ± 9.7 (25.6; 32.8), 33.2	32.1 ± 7.9 (29.2; 35.1), 24.6	29.7 ± 9.4 (26.2; 33.2), 31.5
	Left	34.5 ± 8.5 (31.4; 37.7), 24.5	32.8 ± 7.9 (29.8; 35.8), 24.2	33.6 ± 7.1 (30.9; 36.3), 21.2	30.0 ± 9.1 (26.6; 33.4), 30.4	33.9 ± 7.2 (31.2; 36.6), 21.3	31.2 ± 8.8 (27.9; 34.5), 28.1
	Right	33.1 ± 7.4 (30.3; 35.8), 22.4	30.3 ± 7.9 (27.3; 33.2), 26.1	32.1 ± 8.6 (28.9; 35.3), 26.8	28.4 ± 9.9 (24.7; 32.1), 34.8	31.5 ± 8.1 (28.4; 34.5), 25.7	29.5 ± 9.2 (26.0; 32.9), 31.4
30°	Upper	28.3 ± 13.4 (24.8; 31.7), 47.4	28.4 ± 11.5 (25.4; 31.3), 40.6	29.4 ± 10.8 (26.6; 32.2), 36.8	27.2 ± 10.8 (24.4; 30.0), 39.9	31.±10.1 (28.4; 33.6), 32.6	28.0 ± 11.9 (24.9; 31.0), 42.5
	Lower	33.3 ± 9.2 (31.0; 35.7), 27.6	31.4 ± 8.2 (29.3; 33.5), 26.0	32.4 ± 8.8 (30.1; 34.7), 27.2	30.7 ± 9.7 (28.2; 33.2), 31.7	35.1 ± 9.3 (32.7; 37.5), 26.5	29.9 ± 10.8 (27.2; 32.7), 35.9
	Left	31.9 ± 10.9 (29.1; 34.7), 34.1	29.8 ± 9.8 (27.3; 32.3), 33.0	31.6 ± 9.7 (29.1; 34.1), 30.8	28.3 ± 9.6 (25.8; 30.8), 34.0	33.3 ± 9.7 (30.8; 35.8), 29.2	29.9 ± 10.2 (27.3; 32.6), 34.0
	Right	29.7 ± 12.5 (26.4; 32.9), 42.2	30.0 ± 10.4 (27.3; 32.6), 34.6	30.2 ± 10.2 (27.6; 32.9), 33.8	29.6 ± 11.2 (26.7; 32.5), 37.8	32.8 ± 10.1 (30.2; 35.4), 30.9	28.0 ± 12.4 (24.8; 31.2), 44.3

**TABLE 3:** Statistical results of critical fusion frequency comparisons between runs and sessions for each stimulated eccentricity

Eccentricity	Main effect RUN	Interaction effect RUN × SESSION	Post hoc results for the rest session	Post hoc results for the training session
3°	*F*_2,224_ = 16, *p* < .0001	*F*_2,224_ = 9.9, *p* = .00009	NS	*P*_run1‐run2_ < 0.0001, *P*_run1‐run3_ = 0.0056, *P*_run2‐run3_ = 0.008
15°	NS	*F*_2,224_ = 10, *p* = .00007	NS	*P*_run1‐run2_ = 0.003, *P*_run1‐run3_ > 0.99, *P*_run2‐run3_ = 0.17
30°	*F*_2,464_ = 3.7, *p* = .027	*F*_2,464_ = 9.9, *p* = .00009	NS	*P*_run1‐run2_ = 0.0002, *P*_run1‐run3_ > 0.99, *P*_run2‐run3_ = 0.0002

**FIGURE 3:**
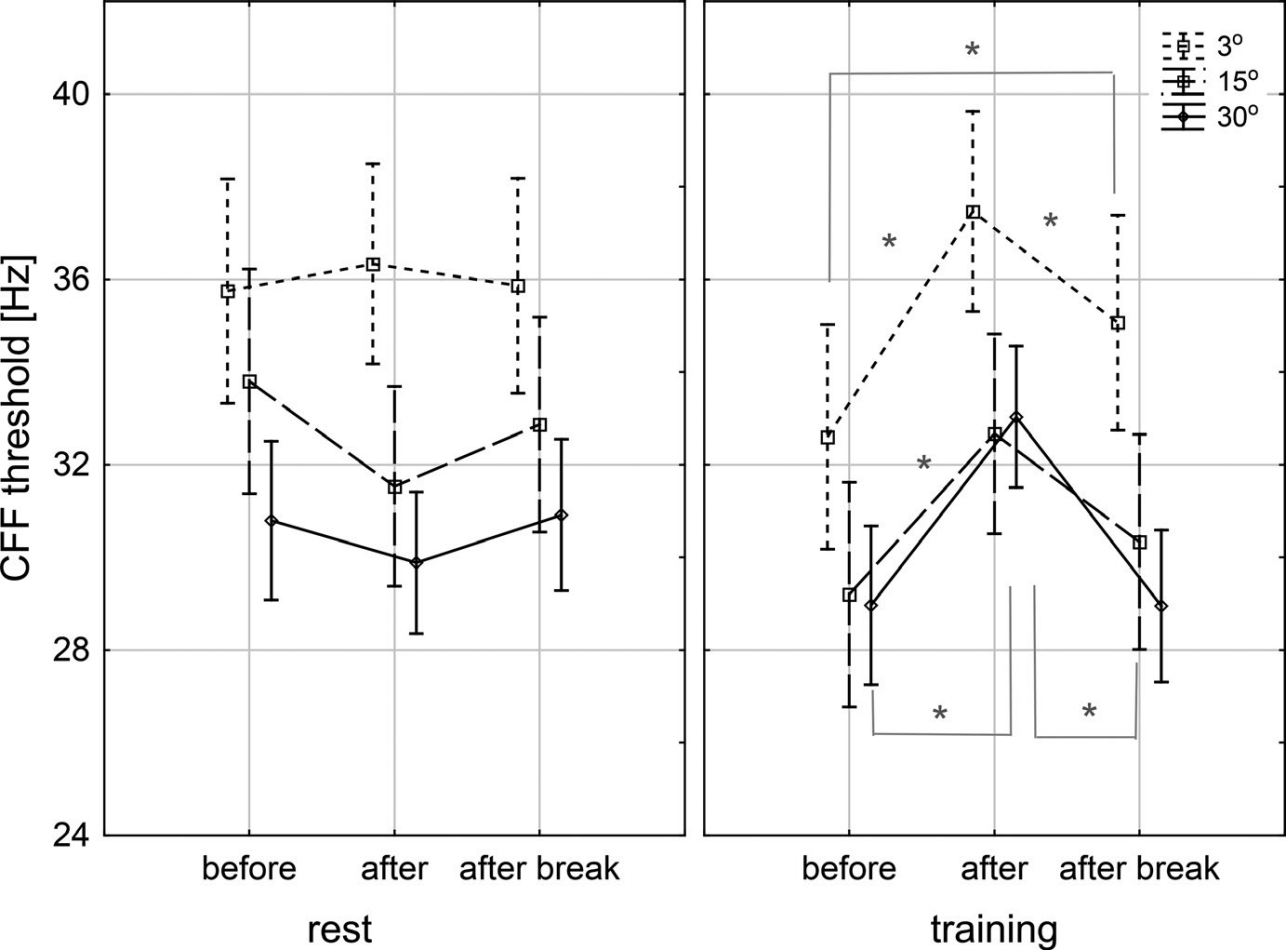
Mean critical fusion frequency (CFF) thresholds before, immediately after the end, and 30 min after the end of rest and training, at 3°, 15°, and 30° eccentricities. *Statistically significant differences, bars – 0.95 confidence level

The most important observations based on the above results are: the lack of changes in CFF between runs during rest for any eccentricity, an increase of CFF after training (run 2) compared to the initial value (run 1) at all eccentricities, and a maintained significantly higher CFF after 30 min break (run 3) compared to the initial value (run 1) only at 3° eccentricity. There were no significant differences regarding VERTICAL VH, or HORIZONTAL VH factor. Mean CFF thresholds before, immediately after the end, and 30 min after the end of training and rest for all three eccentricities, across the whole VF, are presented in Figure 3.

The number of stimuli at 30° eccentricity was twice bigger than at 3° and 15° eccentricities. Though this was carried out on purpose to cover the whole VF evenly, we performed an additional analysis with a reduced number of stimuli at 30° eccentricity to make sure the results are not confounded by the difference in the number of stimuli. To this end, we deleted every other stimulus at 30° eccentricity, leaving only four stimuli at this eccentricity, just like at the other two eccentricities. Statistical analysis gave similar results to that performed on the total number of stimuli. There was marginal main effect RUN (*F*_2,224_ = 3, *p = *.054) and an interaction effect RUN × SESSION (*F*_2,224_ = 3.4, *p* = .037) with post hoc significant differences between run 2 and two other runs (*P*_run1‐run2_ = 0.04, *P*_run1‐run3_ > 0.99, *P*_run2‐run3_ = 0.032).

In addition, since there are findings showing that neurophysiological features differ between men and women, we repeated the analysis including gender factor. Being aware that it reduced statistical power (because there are only seven men and eight women), we treated this analysis as an exploratory part of this work. Including gender factor resulted in the same effects: only RUN (*F*_2,224_ = 16.3, *p* < .0001 and *F*_2,464_ = 3.7, *p = *.028 for 3° and 30° eccentricity, respectively) and RUN × SESSION (*F*_2,224_ = 10.0, *p* = .00009 and *F*_2,464_ = 9.4, *p = *.0001 for 3° and 30° eccentricity, respectively) effects at 3° and 30° eccentricities, and RUN × SESSION (*F*_2,224_ = 9.6, *p* = .0001) effect at 15° eccentricity were significant. No effect containing factor GENDER was significant.

### 3.3 Comparison of CFF differences

In order to compare the changes in the CFF increase after training between the analyzed eccentricities and VHs, differences in CFF thresholds between run 1 and run 2 (CFF_after‐before_) as well as between run 1 and run 3 (CFF_after break‐before_) were calculated (for each stimulated point and each person separately).

#### 3.3.1 Comparison of CFF differences between rest and training sessions

Before we directly compared CFF differences between stimulated eccentricities and between stimulated VHs, we first compared CFF_after‐before_ and CFF_after break‐before_ differences between sessions. The statistical results of this analysis are presented in Table 4. The results showed that CFF_after‐before_ was significantly higher in training than in the rest session for each eccentricity and each VH, except for upper VH at 15° eccentricity. CFF_after break‐before_ differed between training and rest sessions across all the stimuli, for right VH at 3° eccentricity, and marginally at 3° eccentricity.

**TABLE 4:** Statistical results of CFF_after‐before_ and CFF_after break‐before_ differences comparisons between sessions

Grouping factor	Group	CFF_after‐before_ difference	CFF_after break‐before_ difference
None	*—*	*Z*_240_ = 7.0, *p* < .0001	*Z*_240_ = 2.3, *p = *.022
Eccentricity	3°	*Z*_60_ = 4.0, *p = *.00007	*Z*_60_ = 1.8, *p = *.072
	15°	*Z*_60_ = 3.6, *p* = .0003	NS
	30°	*Z*_120_ = 4.7, *p = *.000003	NS
Horizontal hemisphere	Upper	*Z*_120_ = 3.9, *p* = .0001	NS
	Lower	*Z*_120_ = 6.0, *p* < .0001	NS
Vertical hemisphere	Left	*Z*_120_ = 6.0, *p* < .0001	NS
	Right	*Z*_120_ = 3.9, *p* = .00009	NS
Eccentricity × horizontal hemisphere	3° Upper	*Z*_30_ = 2.6, *p* = .009	NS
	3° Lower	*Z*_30_ = 2.9, *p* = .0037	NS
	15° Upper	*Z*_30_ = 1.8, *p* = .075	NS
	15° Lower	*Z*_30_ = 3.4, *p* = .0007	NS
	30° Upper	*Z*_60_ = 2.4, *p* = .017	NS
	30° Lower	*Z*_60_ = 4.2, *p* = .00002	NS
Eccentricity × vertical hemisphere	3° Left	*Z*_30_ = 3.3, *p* = .001	NS
	3° Right	*Z*_30_ = 2.6, *p* = .01	*Z*_30_ = 2.1, *p* = .032
	15° Left	*Z*_30_ = 2.4, *p* = .015	NS
	15° Right	*Z*_30_ = 2.8, *p* = .0059	NS
	30° Left	*Z*_60_ = 4.5, *p* = .000007	NS
	30° Right	*Z*_60_ = 2.0, *p* = .04	NS

Abbreviation: CFF, critical fusion frequency.

The additional analysis with reduced number of stimuli at 30° eccentricity gave similar results to that performed on the total number of stimuli. Significant difference was observed for both CFF_after‐before_ and CFF_after break‐before_ differences between sessions when no grouping was performed. The same pattern of results was observed after grouping factors (eccentricity, horizontal VH, and vertical VH) were included. The only difference was the lack of significant results for 30° Upper and 30° Right groups.

In order to further examine the differences in the changes in CFF after training between eccentricities and VHs, we compared CFF_after‐before_ and CFF_after break‐before_ differences directly between eccentricities and between VHs.

#### 3.3.2 Comparison of CFF differences between eccentricities

**FIGURE 4:**
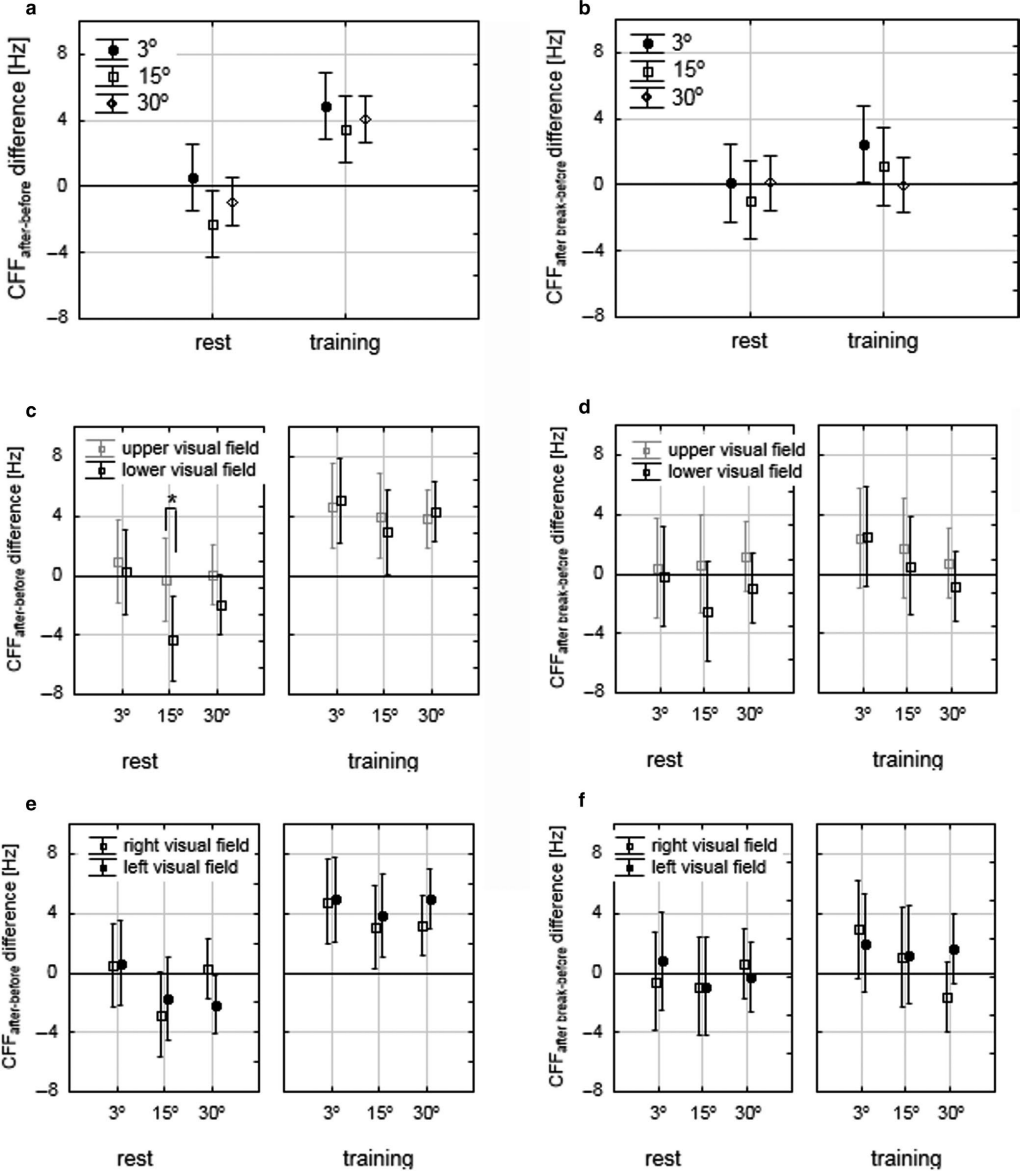
Mean CFF_after‐before_ differences (a, c, e) and CFF_after break‐before_ (b, d, f) differences during rest and training, at 3°, 15°, and 30° eccentricities. Comparison between eccentricities: (a) and (b), comparison between vertical VHs: (c) and (d), comparison between horizontal VHs: (e) and (f). *Statistically significant differences, bars – 0.95 confidence level. CFF, critical fusion frequency; VHs, visual hemifields

The CFF_after‐before_ and CFF_after break‐before_ differences were compared between stimulated eccentricities including SESSION grouping factors, as well as SESSION × HORIZONTAL VH and SESSION × VERTICAL VH. We found a marginally significant difference for CFF_after‐before_ difference between 3° and 15° eccentricities (*H*_2,240_ = 5.6, *p* = .06, post hoc: *p* = .056) in the rest session. When VH factor was taken into account, the only significant result was obtained for CFF_after‐before_ difference between 3° and 15° eccentricities in lower VH (*H*_2,120_ = 7.2, *p* = .027, post hoc: *p* = .028) in the rest session. No significant results were seen in the training session, as well for CFF_after break‐before_ difference in either rest or training session. Mean CFF_after‐before_ and CFF_after break‐before_ differences during rest and training for all three eccentricities are presented in Figure 4a,b.

#### 3.3.3 Comparison of CFF differences between VHs

CFF_after‐before_ and CFF_after break‐before_ differences were comparable between VHs across all eccentricities. The statistical results are presented in Table 5.

**TABLE 5:** Results of comparison of CFF_after‐before_ and CFF_after break‐before_ differences between VHs, across all eccentricities. None of the comparisons was significant

CFF difference parameter	Session	Comparison between horizontal VHs	Comparison between vertical VHs
CFF_after‐before_	Rest	*Z*_120_ = 1.5, *p* = .13	*Z*_120_ = 0.5, *p* = .6
	Training	*Z*_120_ = −0.1, *p* = .91	*Z*_120_ = −1.4, *p* = .17
CFF_after break‐before_	Rest	*Z*_120_ = 1.0, *p* = .30	*Z*_120_ = −0.5, *p* = .64
	Training	*Z*_120_ = 1.2, *p* = .25	*Z*_120_ = −1.1, *p* = .28

Abbreviations: CFF, critical fusion frequency; VHs, visual hemifields.

However, after taking into account the eccentricity, significant results for CFF_after‐before_ difference during rest between horizontal VHs were observed at 15° eccentricity (*Z* = 2.2, *p* = .029). Mean CFF_after‐before_ and CFF_after break‐before_ differences, during rest and training, for horizontal and vertical VHs, and at all three eccentricities are presented in Figure 4c–f.

Again, the additional analysis with a reduced number of stimuli at 30° eccentricity gave similar results to that performed on the total number of stimuli.

#### 3.3.4 Exploratory analysis

Despite the lack of differences of CFF changes in the training session between eccentricities or between VHs, we observed a significant difference in CFF_after‐before_ during rest at 15° eccentricity in lower VH compared to upper VH, which showed that CFF decreased after rest (run 2) at 15° eccentricity in the lower VH, but did not change significantly in the upper VH. Davranche and Audiffren (2004) previously reported a decrease of CFF measured in consecutive sessions when no additional factor was applied and concluded this decrease was caused by the boring nature of the task. In this sense, it is interesting to investigate whether the exercise could compensate for a decrease of CFF induced by mental fatigue in the rest session. Despite we did not observe CFF decrease after rest when absolute CFF thresholds were compared, this investigation could help us to evaluate the potential exercise‐related differences in the CFF increase (as compared to rest) between VFs. Therefore, we conducted an additional, exploratory analysis, and computed two additional parameters for each stimulated point for each person separately, which we then compared between eccentricities and VHs. The first parameter was obtained by subtracting CFF_after‐before_ difference during rest from CFF_after‐before_ difference during training (CFF_(after‐before training)−(after‐before rest)_). Similarly, the second parameter was obtained by subtracting CFF_after break‐before_ difference during rest from CFF_after break‐before_ difference during training (CFF_(after break‐before training)−(after break ‐before rest)_). Each of these two ratios was analyzed with ECCENTRICITY factor (3°, 15°, and 30°) and VERTICAL VH factor (upper, lower), as well as with ECCENTRICITY factor (3°, 15°, and 30°) and HORIZONTAL VH factor (left, right). The inter‐individual variability was much larger than intra‐individual variability induced by exercise effect and we did not obtain significant differences in this analysis, though two trends were observed. The first one was a trend of higher value of CFF_(after‐before training)−(after‐before rest)_ parameter in lower (6.1 ± 10.9) compared to upper VH (3.9 ± 13.0), especially at 15° eccentricity (7.2 ± 12.2 and 4.3 ± 10.1 for lower and upper VH, respectively). The second one was a trend of higher value of CFF_(after‐before training)−(after‐before rest)_ and CFF_(after break‐before training)−(after break‐before rest)_ parameters in left (7.1 ± 14.4 and 1.9 ± 15.8, respectively) compared to right (2.9 ± 13.2 and −2.2 ± 17.1, respectively) VH at 30° eccentricity.

## 4 DISCUSSION

The goal of this work was to evaluate the duration of the effect of an acute submaximal physical exercise on the temporal resolution of the human visual system. To this end, CFF was analyzed in the group of 15 participants before, immediately after the end, and 30 min after the end of indoor cycling (training session). The results were then compared to the results of the rest session. First, the changes in CFF values throughout rest and training sessions (between runs, i.e., consecutive measurements) will be discussed to answer the question of whether (and how) CFF changed after this type of exercise in relation to rest, and how long the changes maintained. Then, a comparison of the observed CFF changes in rest and training sessions between eccentricities will be discussed to answer the question of whether these changes were similar or different between central and peripheral areas of VF. Last, a comparison of the observed changes in CFF in rest and training sessions between VHs will be discussed to answer the question of whether the observed changes differed between upper and lower, as well as between left and right VHs.

Many studies have presented alterations of cognitive processes due to acute physical exercise (Davranche & Audiffren, 2004; Davranche & Pichon, 2005; Hanson et al., 2018; Krebs et al., 1989; Lambourne & Tomporowski, 2010). CFF increase is regarded as an increase in cortical arousal, sensory responsiveness, information processing or executive functions, while CFF decrease is an indicator of a reduction of the efficiency of the system to process information (Davranche & Pichon, 2005). However, a discrepancy in the obtained results due to a wide spectrum of types, length, intensity, and modality of exercise used in these studies, as well as other factors, like initial fitness level has been discussed in the literature (Hanson et al., 2018; Simonson & Brozek, 1952). This discrepancy makes it difficult to compare the results, and more importantly, draw long‐term conclusions. What is even more interesting, there are very few studies evaluating the duration of the observed effects after the termination of exercise. This is especially important when stimulatory effects of exercise on high‐level cognitive functions are investigated in terms of improvement of cognition, training programs or sport strategies. To our best knowledge, there are no studies showing the duration of the stimulatory effect of acute physical exercise on temporal visual sensitivity by means of CFF.

Moreover, Lambourne and Tomporowski (2010) in their meta‐analysis of the effects of exercise‐induced arousal on cognitive performance, pointed out that many studies on the relation between exercise and cognition do not properly control for possible confounds and therefore may overestimate their effect sizes. To eliminate the biological and psychophysiological confounding factors, we controlled for the age, body weight and height, initial HR, initial pupil size, and initial mental fatigue level. Correlations of the biological factors with CFF were not significant (Table 1), ensuring the proper control for these factors. Moreover, we performed our experiment among young adults who practice sport on a recreational level, since the effect size was expected to be higher in this group than in experienced cyclists. We used a submaximal workload to ensure obtaining the neurocognitive benefits of exercise without crossing the threshold when physical fatigue would occur (Hanson et al., 2018). Finally, we chose indoor cycling, as this mode was shown to produce larger effect sizes when compared to the treadmill (Lambourne & Tomporowski, 2010), due to differences in cortical activation specific to the musculature involved in each task.

### 4.1 Duration of the effect of exercise on CFF

Our results showed that at all examined eccentricities (3°, 15°, and 30°), CFF thresholds increased immediately after the end of the training, while no differences were observed during rest (Table 3; Figure 3). Interestingly, this increase maintained significantly in fovea (3° eccentricity) 30 min after the end of the training (Table 3; Figure 3). CFF in perifovea (15°) was somewhere between the values before and immediately after, but this difference was not significant. CFF in the midperiphery (30°) decreased 30 min after the end of the training to reach similar values as before the training. Our results suggest that the stimulatory effect of an acute, submaximal exercise, though observed at every analyzed eccentricity, differed in duration in relation to the distance from the fovea.

The observed CFF changes might have been confounded with the changes in pupil size due to an increased area of illumination after training. Therefore, we compared pupil size between runs in both sessions and did not find any differences (no main effect RUN [*F*_2,56_ = 0.8, *p* = .44], nor an interaction effect RUN × SESSION [*F*_2,56_ = 0.7, *p* = .48]). These results showed that the exercise‐induced changes in CFF were not due to a change in pupil size. We also took into account a possible direct relationship of CFF with HR. As was expected, HR did not change during rest. It increased significantly after training and then got back to normal 30 min after the end of the training (RUN [*F*_2,56_ = 191, *p* < .0001], and an interaction effect RUN × SESSION [*F*_2,56_ = 235, *p* < .0001], post hoc *P*_run1‐run2_ < 0.0001, *P*_run2‐run3_ < 0.0001, *P*_run1‐run3_ > 0.99 in the training session and *P*_run1‐run2_ = 0.64, *P*_run2‐run3_ > 0.99, *P*_run1‐run3_ > 0.99 in the rest session). Therefore, the maintained increase of CFF 30 min after the end of the training can not be explained by the direct increase in HR.

The possible explanation of the stimulatory effect of an acute exercise can be discussed on the ground of retinal anatomy, hormonal and vascular changes in the body, and higher‐order cognitive changes.

On the retinal level, CFF has been linked to ganglion cell layer thickness and macular pigment density (Schrupp et al., 2009). Beyond the retina, CFF has been used to provide a measure of the intactness and efficacy of the underlying neural pathways and related visual processing which begins at the lateral geniculate nucleus (LGN), where it is separated into the magnocellular (M) and parvocellular (P) pathways (Schrupp et al., 2009). The magnocellular pathway is assumed to be involved in the perception of both motion and flicker (Balestra et al., 2018).

There are several mechanisms operating on a hormonal and vascular level, which have been suggested for exercise‐related improvement in the processing of visual stimuli, such as neurogenesis and synaptogenesis through increased production of brain‐derived neurotrophic factor, augmented neurotransmitter levels and effectiveness, peripheral mechanisms, including the release and transport of circulating catecholamines, an increase in cerebral blood flow, and stimulation of the neuroendocrine system by the modulation of the sympathoadrenal system and hypothalamic–pituitary–adrenal axis (Al‐Nimer & Al‐Kurashy, 2007; Balestra et al., 2018; Hanson et al., 2018; Simonson & Brozek, 1952).

The changes in the higher‐order cognitive functions have been discussed in terms of neural arousal, decision‐making, response inhibition, and cortical integration by means of white matter tracks including feedforward and feedback connections that link hierarchical areas in the brain and horizontal connections within each area (Hanson et al., 2018; Schrupp et al., 2009; Woods & Thomson, 1995).

Lambourne and Tomporowski (2010) in their meta‐analysis based on 29 studies that provided pre‐ and post‐exercise cognitive measures, showed that participants' cognitive performance improved when tested after exercise, which confirmed the earlier predictions that metabolic recovery occurs gradually and the heightened level of arousal during this period facilitates cognitive function. At the same time, the authors emphasized the lack of studies presenting changes following the termination of exercise and the need for investigating short‐term after‐effects.

Our findings fill the gap in this area. We suggest that the stimulatory effect of an acute indoor cycling exercise on CFF maintain even 30 min after the end of the exercise. However, the duration of the effect in our study depended on the eccentricity and was the most pronounced when foveal retina was stimulated.

### 4.2 The effect of eccentricity

The differences in the duration of the effect between eccentricities that we observed when CFF were compared between runs were further investigated using CFF_after‐before_ and CFF_after break‐before_ differences. CFF_after‐before_ difference was significantly higher for training than for the rest session across all eccentricities, as well as for each eccentricity. However, CFF_after break‐before_ difference was significantly higher for training than for the rest session across all eccentricities, for right VH at 3° eccentricity, and marginally higher at 3° eccentricity (Table 3). Though the difference of CFF_after break‐before_ differences between eccentricities in the training session did not reach significance (Figure 4b), the mean CFF_after break‐before_ difference decreased from the fovea toward the midperiphery, being above 0 in fovea and perifovea, while reaching 0 in midperiphery. These results suggest a different duration of the effect between eccentricities. It seems that in fovea, an increased CFF threshold remained 30 after the training, in perifovea it decreased, but still was higher than before the training, while in midperiphery it equalized with the threshold recorded before the training.

Changes in CFF sensitivity with eccentricity on the retinal level have been related to changes in photoreceptor dimensions (Anderson & Vingrys, 2002). These differences may also be connected to the different distributions of photoreceptors along retina which strongly depends on the eccentricity. Cones are localized mainly in the center and rods' concentration is maximal around 15–20° eccentricity and then decrease toward far periphery. Since CFF_after break‐before_ difference was the highest at 3° eccentricity, that is where cones reach their maximal concentrations, and the lowest at 30° eccentricity, the explanation of our results based on photoreceptors differences may be that an acute physical exercise in the form of indoor cycling has a stimulatory effect on both types of photoreceptors, but the effect remains longer in cones. However, the extrafoveal retina has been regarded to be more sensitive to the detection of motion and flicker than fovea, due to a greater contribution to the magnocellular pathways (Schrupp et al., 2009). Interestingly, we did not observe a greater impact of exercise on the extrafoveal area compared to fovea, which should be expected.

The possible explanation of our results is that training improved information processing and cognitive functions rather than retinal sensitivity per se. It has been previously suggested that CFF alteration under abnormal conditions, such as physical activity, more likely depend on brain physiology than eye hemodynamics (Balestra et al., 2018; Powell, 1982; Simonson & Brozek et al., 1952; Vani et al., 1997; Woods & Thomson, 1995). This view was supported by the measures of both temporal and spatial summation. The optic nerve up to and including the LGN have been demonstrated to reflect higher rates of discrete light inputs than were actually cognitively perceived. However, no strong conclusion can be made without further investigating the observed effect.

### 4.3 The effect of vertical VF

Vertical asymmetries between visual signal processing in healthy humans have been reported and the contribution of both spatial attention and structural organization of the visual system, including the anisotropies within the retina and the cortical retinotopic areas, has been discussed (Cheng et al., 2019; Curcio & Allen, 1990; Levine & McAnany, 2005; Rezec & Dobkins, 2004; Silva et al., 2014; Tyler, 1987; Zito et al., 2016). Lower VH has been shown to advantage for low‐level stimuli (contrast sensitivity, space and motion processing, light sensitivity in orientation, discrimination, detection, localization, and visual search tasks), whereas upper VH benefits for higher level visual processing, such as complex objects and shapes or face processing (Rezec & Dobkins, 2004; Silva et al., 2014; Zito et al., 2016).

The attentional factors may be connected to the activation of dorsal and ventral visual streams. Local processing of the shape and color of the object is believed to be selective to parvocellular pathways, while global processing of the space and motion to magnocellular pathways (Cheng et al., 2019; Zito et al., 2016). Moreover, since V1 receives inputs from both pathways, any observed asymmetries are more likely linked to the later stages of visual processing (Zito et al., 2016).

Other explanation was attributed not to attention, but to visual constraints (Levine & McAnany, 2005; Zito et al., 2016). According to the ecological theory of Previc (1990), processing differences between the vertical hemifields are related to the distinction between near (peripersonal) and far (extrapersonal) space, which are biased toward the lower and upper VHs, respectively.

In terms of the aforementioned mechanisms, flicker stimuli are supposed to be processed more efficiently in the lower VH compared to upper VH. Therefore, we should expect higher absolute CFF thresholds in the first examination (run 1, i.e., before the training or rest) in the lower than the upper VH. Despite the fact that in the first step of our analysis, the VERTICAL VH factor was not significant, the CFF thresholds were systematically higher in lower than upper VH at 30° eccentricity, for both sessions (Table 2), what is in the agreement of previously presented asymmetries.

When CFF_after‐before_ and CFF_after break‐before_ differences were analyzed, we did not observe differences between vertical VHs in the training session. Interestingly, the magnitude of CFF_after‐before_ difference was higher (with a negative sign) for lower than for upper VH during rest, especially at 15° eccentricity, where the difference was significant (Figure 4c). The fact that CFF decreased during rest might be explained by the mental fatigue due to the repetitive nature of the task, and habituation phenomena, which has been observed previously (Davranche & Audiffren, 2004). Though we did not observe a significant drop of the absolute CFF threshold during rest between runs (Table 3; Figure 3), the difference in CFF_after‐before_ may suggest that the habituation process runs differently in these two VHs.

The question then arises whether exercise could compensate for CFF decrease induced by mental fatigue in the rest session. To answer this question, we performed an exploratory analysis, where we compared CFF_(after‐before training)−(after‐before rest)_ as a difference between CFF_after‐before_ difference during training and CFF_after‐before_ difference during rest, as well as CFF_(after break‐before training)−(after break ‐before rest)_) as a difference between CFF_after break‐before_ difference during training and CFF_after break‐before_ difference during rest. Though we did not obtain significant differences in this analysis, were observed a trend of higher value of CFF_(after‐before training)−(after‐before rest)_ parameter in lower compared to upper VH, especially at 15° eccentricity. This result suggests that acute exercise may have compensated rest‐induced CFF lowering in lower VH, but these results should be interpreted with caution. The mechanisms responsible for the VH asymmetries cannot be explained based on our study. However, implementation of EEG and ERP (event‐related potentials) methods in our future work will help us to investigate the possible attentional factor in the temporal processing of visual stimuli.

### 4.4 The effect of horizontal VF

Cortex asymmetrical responsiveness to stimuli presented in left or right VH has also been discussed (Powell, 1982; Zito et al., 2016). Studies in patients suggested that there are two independent groups of neurons that are responsive to stimuli in only one VH. However, Zito et al. (2016) in their study on content‐dependent perceptual asymmetries in different regions of the VF among healthy participants did not confirm this pattern in any of the proposed subtasks (three visual tests involving the perception of shapes, orientation, and motion).

Our results are in agreement with Walter et al. (2015) who studied sustained splits of attention within and across VHs. The authors did not observe differences between left and right VHs in early visual processing and behavioral performance. Silva et al. (2014) also did not observe left/right hemifield asymmetry in the case of low spatial and high temporal frequency in the contrast sensitivity study.

It seems that the strong interconnectivity of the two hemispheres (Carlei & Kerzel, 2017) may compensate for all the possible asymmetries in CFF. Our results suggest that the differences in the perception of visual stimuli between left and right VFs observed in the literature are more likely task‐related and manifest in specific higher‐order information processing (e.g., attention reorienting, detection or competition) rather than the temporal sensitivity (Carlei & Kerzel, 2017; Cheng et al., 2019; Corbetta & Shulman, 2011; Powell, 1982; Wright et al., 2016). However, based on the trend of higher value of CFF_(after‐before training)−(after‐before rest)_ and CFF_(after break‐before training)−(after break ‐before rest)_ parameters in left compared to right VH at 30° eccentricity, which we observed in our exploratory analysis, abnormal conditions (such as exercise) may act differently on these two VHs. These results need further investigation. The use of EEG and ERP to measure potential cognitive exercise‐related hemispheric asymmetries might be helpful.

## 5 CONCLUSIONS

The goal of this study was to evaluate the effect of acute, submaximal physical exercise (indoor cycling) on the temporal resolution of the human visual system, using CFF over time, with regards to eccentricity and VHs. During rest, CFF did not change significantly, but we observed an increased CFF immediately after training at all studied eccentricities. The stimulatory effect of training remained significant 30 min after the end of the training in fovea, but was negligible in midperiphery. Our results suggest that an acute, moderate‐intensity cycling improved the temporal resolution of the vision system in non‐experienced cyclists, with the duration of the effect depending on eccentricity. These findings are very important in relation to a broad spectrum of reported studies, indicating the improvement of cognitive processes after physical activity. When VHs were compared, greater decrease of CFF during rest was observed for lower than for upper VH, especially at 15° eccentricity. We suggested a possible compensating effect of an acute exercise on this lowering of CFF during rest in the lower VH, which needs further investigation. However, since this result was not statistically significant, it should be interpreted with caution. The possible mechanisms, which underlie our results, will be further investigated by means of CFF extended by EEG and ERP measurements.

### 5.1 Further investigations

This study gave us the opportunity to set a direction for future investigations of the effect of acute physical exercise on the temporal resolution of the human visual system. Further investigation on bigger sample size will elucidate possible differences of this effect between eccentricities and between VHs, observed in this study. It is also interesting to compare the stimulatory effect of submaximal training between non‐experienced (like in this study) with experienced sportsmen. Cavalade et al. (2015) in their study did not find a CFF lowering during and after a skydive jump among experienced skydivers, which suggested that hypoxia, which is known to induce autonomic shifts in cardiovascular regulation by the ANS, was well tolerated by the experienced skydiver and did not affect the ANS (Cavalade et al., 2015). A comparison of CFF changes after acute cycling among experienced and non‐experienced volunteers could help to dissociate whether it is due to a general effect in body metabolism or rather a neurophysiological change. In addition, we want to incorporate EEG and ERP methods, which will more precisely indicate the potential effect of training on neurophysiological changes in visual cortex and neural arousal, especially in the light of previous findings of CFF thresholds being significantly higher in retinal than in cortical cells, but also related to other brain regions linked to the processing of a flickering stimulus (Balestra et al., 2018; Brown et al., 2018; Eisen‐Enosh et al., 2017; Hanson et al., 2018; Schrupp et al., 2009; Wells et al., 2001).

### 5.2 Study advantages and limitations

The strength of this study lies in a very carefully designed, prepared, and conducted a controlled experiment. Since CFF is a subjective measure, based strongly on the cooperation of the experimenter with the participant and the proper understanding of the measurement, the volunteers participated in three separate sessions. The goal of the demonstration was to introduce the participants to the measurement and to minimize the number of errors during the following sessions (what resulted in fulfilling the maximal error criterion by all participants). Then a training session was conducted, which included a cycling exercise and three CFF measurements (before, immediately after the end of exercise and 30 min after the end of exercise). Rest session, which also included three CFF measurements, was performed on a different day. The length of the training session was set individually and depended on the time needed to obtain 75% of HR_max_, which allowed us to obtain the same workload for every participant. The rest session allowed us to control for possible effects of consecutive CFF measurements. The fact that it was performed on a different day, which was later than the training day, allowed us to set the timing of CFF measurements the same as the timing of CFF measurements during the training session. The length of the CFF examination is also crucial and there must be a compromise between using enough stimuli to cover the whole VF and examination being short enough to not cause visual or mental fatigue. Therefore, we used a custom set of 24 stimuli to cover both central and peripheral VF, but coarse enough so that participants would not have problems with completing the task without errors. Moreover, pupil diameter measurements allowed us to control for possible confound of differences in CFF resulted from different retinal illumination areas. We also controlled for other possible biological confounds, such as differences in age, body mass, fatigue level or initial HR.

The limitation of this study is a small sample size, which may have resulted in not obtaining all possible effects significantly. However, this sample size is comparable (and often higher) than those used in previous research on the influence of physical exercise on CFF (Lambourne & Tomporowski, 2010).

The second limitation is the use of a 220‐age estimate of HR_max_. This equation is widely used in clinical medicine and physiology, by which health care professionals assess exercise effort. However, recent studies indicate this measure may not accurately estimate HR_max_. Other methods have been suggested, such as different estimation formulas derived by Tanaka et al. (2001) and Arena et al. (2016), as well as the Borg RPE scale, and they will be considered in our future studies.

Higher mean CFF values observed for “before rest” compared to “before training” conditions (Figure 2) may have several causes, including “residuals” from the training session effect, practice effect or interindividual differences. Due to the fact that the time interval between training and rest sessions was 10 ± 9 days, the first effect can be excluded. To investigate whether this difference is truly systematic for all subjects, or it rather comes from individual differences, we plotted a scatterplot of CFF_before rest_ − CFF_before training_ differences for every stimulated point, for every participant. This plot showed us two participants, who were outliers. We, therefore, reran the analysis after excluding those two subjects and observed all the results were the same as our original results. Though this suggests individual differences and differences in a psychophysiological state of the participants as a cause of difference in initial CFFs between training and rest sessions, we will keep in mind this issue in our future work and put emphasis on test‐retest validity.

## CONFLICTS OF INTEREST

The authors report no conflicts of interest.

## AUTHOR CONTRIBUTION

The experiment was performed at the Institute of Biomedical Engineering, Faculty of Science and Technology, University of Silesia in Katowice, Poland. KM conceived and designed the work, analyzed and interpreted the data for the work, and drafted the manuscript. AG and AW collected the data and revised the manuscript critically for important intellectual content. All authors approved the final version of the manuscript and agree to be accountable for all aspects of the work in ensuring that questions related to the accuracy or integrity of any part of the work are appropriately investigated and resolved. All persons designated as authors qualify for authorship, and all those who qualify for authorship are listed.

## Data Availability

We will make the data associated with our paper available.
